# Modulation of histone H3K4 dimethylation by spermidine ameliorates motor neuron survival and neuropathology in a mouse model of ALS

**DOI:** 10.1186/s12929-022-00890-3

**Published:** 2022-12-20

**Authors:** Seung-Hye Choi, Ali Yousefian-Jazi, Seung Jae Hyeon, Phuong Thi Thanh Nguyen, Jiyeon Chu, Sojung Kim, Suhyun Kim, Hannah L. Ryu, Neil W. Kowall, Hoon Ryu, Junghee Lee

**Affiliations:** 1 K-Laboratory, Brain Science Institute, Korea Institute of Science and Technology (KIST), Seoul, 02792 South Korea; 2 KIST School, Division of Bio-Medical Science & Technology, University of Science and Technology (UST), Seoul, 02792 South Korea; 3 Integrated Biomedical and Life Science Department, Graduate School, Korea University, Seoul, 02841 South Korea; 4 Department of Neurology, Boston University Alzheimer’s Disease Research Center, Boston University School of Medicine, Boston, MA 02118 USA; 5 VA Boston Healthcare System, 150 S. Huntington Avenue, Boston, MA 02130 USA

**Keywords:** Amyotrophic lateral sclerosis, LSD1, Spermidine, Histone H3K4 dimethylation, Motor neuron

## Abstract

**Background:**

Amyotrophic lateral sclerosis (ALS) is a fatal neurodegenerative disorder characterized by progressive paralysis due to motor neuron degeneration. It has been proposed that epigenetic modification and transcriptional dysregulation may contribute to motor neuron death. In this study, we investigate the basis for therapeutic approaches to target lysine-specific histone demethylase 1 (LSD1) and elucidate the mechanistic role of LSD1-histone H3K4 signaling pathway in ALS pathogenesis.

**Methods:**

In order to examine the role of spermidine (SD), we administered SD to an animal model of ALS (G93A) and performed neuropathological analysis, body weight, and survival evaluation.

**Results:**

Herein, we found that LSD1 activity is increased while levels of H3K4me2, a substrate of LSD1, is decreased in cellular and animal models of ALS. SD administration modulated the LSD1 activity and restored H3K4me2 levels in ChAT-positive motor neurons in the lumbar spinal cord of ALS mice. SD prevented cellular damage by improving the number and size of motor neurons in ALS mice. SD administration also reduced GFAP-positive astrogliogenesis in the white and gray matter of the lumbar spinal cord, improving the neuropathology of ALS mice. Moreover, SD administration improved the rotarod performance and gait analysis of ALS mice. Finally, SD administration delayed disease onset and prolonged the lifespan of ALS (G93A) transgenic mice.

**Conclusion:**

Together, modulating epigenetic targets such as LSD1 by small compounds may be a useful therapeutic strategy for treating ALS.

**Supplementary Information:**

The online version contains supplementary material available at 10.1186/s12929-022-00890-3.

## Introduction

Amyotrophic lateral sclerosis (ALS) is a devastating neurodegenerative disease characterized by a loss of upper and lower motor neurons resulting in progressive muscular atrophy and paralysis. ALS has an annual incidence of 1–2 per 100,000 and is usually fatal within 2–5 years of diagnosis [1]. Sporadic ALS (sALS) makes up the vast majority, but about 5–10% of instances are familial, whose clinical and pathological features are indistinguishable from those of sALS. Notably, 25% of familial ALS (fALS) cases are due to the missense mutations of SOD1 [2]. Transgenic mice expressing either G93A or G37R human SOD1 mutations with elevated levels of SOD1 activity, or G85R SOD1 mutations with protein and activity levels essentially equal to that of endogenous levels, develop progressive hind limb weakness, muscle wasting, and neuropathological sequelae similar to those observed in both sALS and fALS patients [3–6]. Beneficial therapeutic results have been observed in transgenic ALS mice, but have had minimal success in human clinical trials [7–10]. Thus, a better understanding of the molecular events that cause motor neuronal death in ALS may lead to new therapeutic targets.

Given that mutations of the important antioxidant enzyme SOD1 are a cause of fALS, it is not unreasonable to consider oxidative stress as one of the key roles in disease pathogenesis. Previous studies have shown that oxidative stress regulates the epigenetic modifications [11, 12]. Strivas et al. demonstrated that oxidative stress-mediated epigenetic changes play a critical role in memory processes during aging in several neurological disorders and their recovery through antioxidant treatment [13]. Indeed, many studies have documented oxidative damage in both postmortem human ALS tissues and transgenic animal models [3, 14–16]. Since, a direct relationship between SOD1 mutations and oxidative injury has not been established yet, and how these mutations cause ALS remains a hotly debated research topic [17, 18]. Abnormalities in glutamate regulation have also been identified in ALS, suggesting excessive synaptic glutamate and oxidative stress in the initiation or propagation of motor neuron loss [19–21]. Our previous study has shown that l-arginine protects motor neurons from glutamate-induced toxicity in vitro and prolongs the survival of ALS (G93A) transgenic mice [22]. l-arginine, a substrate for both arginase-1 and nitric oxide synthase, is a precursor for polyamine synthesis [23]. Polyamines are known to be potent inhibitors of lysine-specific histone demethylase-1 (LSD1), a critical enzyme in regulating histone and non-histone protein methylation [24]. Previous studies showed LSD1 can remove dimethyl groups from the fourth positions on histone 3 protein (H3K4me2) associated with gene repression [25, 26]. LSD1 demethylates H3K4me2 through an oxidative reaction that leads to the decrement of the protein-bound flavin adenine dinucleotide (FAD) cofactor and the production of H_2_O_2_ [25–27]. Moreover, Huang et al. demonstrated that LSD1 may play a role in cell death by mediating p53 demethylation, thereby inhibiting its interaction with its co-activator 53BP1 to induce apoptosis [28].

Despite attempts to prevent disease progression, no cure or effective therapy is currently available for ALS. A better understanding of LSD1 and its role in regulating gene expression can aid in discovering novel strategies in restoring inappropriately silenced genes. LSD1 belongs to a growing number of transcriptional complexes implicated in ALS pathogenesis. Therefore, there have been increased efforts to identify or design LSD1 inhibitors that could function as therapeutic agents [29–32]. From this perspective, the evolutionary and functional similarities with monoamine oxidases (MAOs) and polyamine oxidases (PAOs) have proven to be particularly insightful. Researchers have been testing known inhibitors of these amine oxidases against LSD1 [33]. Although LSD1 is uninhibited by most, those that do display significant levels of reactivity. Several research groups have been studying clinically approved MAO inhibitors, a well-known drug target for neurological disorders [34, 35]. Several MAO inhibitors function through a mechanism-based mode that generates a covalent adduct with FAD cofactor. Indeed, an H3 peptide bearing a propargyl-modified Lys4 reacts with the flavin moiety [36, 37]. As guanidines have been shown to inhibit both spermine oxidase (SMO/PAOh1) and other polyamine oxidases, we sought whether arginine supplementation and polyamines (spermidine and spermine) administration will effectively inhibit the endogenous expression or activity of LSD1 and whether the subsequent modulation of LSD1 could lead to the protection of motor neurons and improve neuropathology of ALS. However, the mechanism of LSD1 induction in the pathogenesis of ALS and therapeutic modulation of LSD1 activity in this neurodegenerative condition has not been investigated.

Therefore, our study aimed to investigate the basis for therapeutic approaches to target LSD1 and elucidate the mechanistic role of LSD1-H3K4 signaling pathway in ALS pathogenesis. Herein, we found that LSD1 protein is induced and H3K4me2 level is markedly decreased in both cellular model of ALS [mSOD1 (G93A)] and animal model of ALS [mSOD1 (G93A)]. Moreover, LSD1 is increased in NSC-34 cells treated with hydrogen peroxide to produce oxidative stress and in spinal motor neurons of ALS (G93A) mice, supporting the potential role of LSD1 in ALS pathogenesis. We further examined the therapeutic effect of spermidine (SD) on the modulation of LSD1, neuropathology, behavior, and survival rate of ALS transgenic mice.

## Methods

### Animals

Male transgenic ALS mice of the G93A H1 high-expresser strain (Jackson Laboratories, Bar Harbor, Maine) are bred with females with a similar background (B6/SJLF1). Offspring were genotyped using a PCR assay on tail DNA. To ensure homogeneity of the cohorts tested, we have developed a standardized method to select mice. Mice were randomized from 24 litters all within 4 days of age from the same ‘f’ generation removed from the founding mice in our colony. Body weights were taken at 20 days and mice were equally distributed according to weight within each experimental cohort. Mice under 8 g at 20 days of age were excluded from the experiments. Only male mice were used in the treatment studies since there are gender differences in survival in the G93A transgenic ALS mouse model [38].

### Spermidine administration

SD and saline were separately administered (5 mg/kg, I.P. injection) to groups of 6 wildtype and ALS (G93A) mice from 30 to 120 days of age 4 times per week. Injection begins 30 and 70 days of age, before the occurrence of symptoms in G93A mice. Control groups of G93A mice were injected with saline only.

### Histopathological evaluation

Groups of 6 animals from saline and SD treatment paradigm were deeply anesthetized and transcardially perfused with 4% PFA at 120 days of age. Serial-cut lumbar spinal cord tissue-sections (*n* = 10), from L3–L5 spinal cord segments were used for neuronal analysis. Spinal cord tissue sections were stained for Nissl substance and immunostained for glial fibrillary antigen protein (GFAP), LSD1, H3K4me2, SMI32, ChAT, etc., as previously described [39].

### Motor performance and behaviors

#### Rotarod test

The rotarod (Panlab, Harvard) was used to assess motor performance of mice. All mice were familiarized with the rotarod apparatus for 5 days prior to testing. Mice were subjected to the rotarod test with 3 trials per day. Mice were placed on the rod, and the apparatus was set to accelerating mode (4–40 rpm in 200 s). The latency to fall with upper limit of 200 s per trial was measured [40].

#### Wheel test

The gait analysis was performed using a wheel test. All mice were familiarized with the wheel apparatus with exactly the same conditions as the real test right before test. Each mouse was placed inside of the wheel and the apparatus was to accelerating mode (4–15 rpm in 180 s). Both the bottom and front views of the wheel test were recorded by two cameras. For the footprint analysis, the hindlimbs and forelimbs of mice were dyed with different colors using non-toxic animal marking stick. The gait analysis was simulated by the EthoVision XT software (Noldus). Seven or eight continuous strides were analyzed for each mouse [41].

### Clinical assessment and survival

Both body weight and survival were measured throughout the study. Body weights were recorded twice a week at the same time of day. G93A mice were assessed twice daily (mid-morning and late afternoon) for morbidity and mortality.

### Immunofluorescence staining and confocal microscopy

The tissue sections were incubated with blocking solution containing 0.3% Triton X-100, 5% bovine serum albumin, and 3% goat serum for 1 h followed by incubation with SMI32 mouse monoclonal antibody (1:200 dilution, Abcam), anti-LSD1 rabbit monoclonal antibody (1:200 dilution, Cell Signaling), anti-H3K4me2 rabbit monoclonal antibody (1:400 dilution, Cell Signaling), and ChAT antibody (BD Biosciences) overnight at 4 °C. After three washes with PBS, the cells were incubated for 1 h with FITC-conjugated goat anti-mouse IgG antibody (1:200 dilution; Vector Laboratories, Burlingame, CA) and Cy3-conjugated goat anti-rabbit IgG antibody (1:200 dilution; Jackson Laboratories). All antibodies were diluted in PBS. The slides were washed three times with PBS and mounted with fluorochrome mounting solution (Vector Laboratories). Moreover, images were taken by a spinning confocal microscopy (Olympus DSU, Tokyo, Japan), and the size of motor neuronal cell body was analyzed by AQI-X-COMBO-CWF program (Media cybernetics Inc. Bethesda, MD). Briefly, we took a series of 50 confocal layers representing fluorescence data from the ventral horn, then analyzed all layers by the software. Control experiments were performed in the absence of primary antibody.

### Measurement of LSD1 activity

LSD1 activity was determined from spinal cord lysates that had been administered with SD or vehicle (saline). The spinal cord tissues were minced and homogenized in PBS containing protease inhibitors (2 µM PMSF, 2 µg/ml pepstatin A, and 10 µg/ml leupeptin) with 23 full strokes by Dounce homogenizer on ice. The lysates were centrifuged at 4 °C at 3000 rpm for 10 min. The pellet (nuclear fraction) was resuspended in 350 μm NaCl (high salt) buffer with protease inhibitors to lyse nuclear membranes, forcing DNA into solution. The nuclear lysates were centrifuged at 15,000 rpm for 20 min at 4 °C. The supernatant was aliquoted and its protein concentration was measured by Bradford assay. The 10 µg of nuclear extract was reacted according to the protocol from EpiQuik Histone Demethylase LSD1 Activity Assay Kit (Epigentek Group Inc., Brooklyn, NY, USA). The LSD1 standards were included for the quantification of LSD1 activity. Finally, the endpoint result of LSD1 activity was measured and read on a 96 well fluorescence microplate reader at 530_EX_/590_EM_.

### NSC-34 cell lines culture

NSC-34 mouse motor neuron-like cell lines transfected with pCI-neo expression vector containing human WT-SOD1 and mSOD1(G93A) were maintained in Dulbecco’s modified Eagle’s medium supplemented with 10% (v/v) fetal bovine serum, 100 U/ml penicillin and 0.1 mg/ml streptomycin. Cells were kept in a humidified incubator at 37 °C under 5% CO_2_. Cells were sub-cultured in 60 mm dishes at a density of 1 × 10^6^ cells/well. After 80% confluence, cells were treated with 100 µM hydrogen peroxide (H_2_O_2_) for 12 h. For immunostaining experiments, cells were seeded in 6-well plates containing 13 mm round coverslips at a density of 5 × 10^5^ cells/well. For drug treatment, NSC-34/WT-SOD1 cells and NSC-34/mSOD1(G93A) cells in six-well plates were treated with SD (5 µM) for 24 h.

### RNA isolation and quantitative real–time PCR (qPCR)

qPCR was performed with ABI PRISM 7700 Sequence Detection System Instrument and software (Applied Biosystems, Foster City, CA, USA), using the manufacturer’s recommended conditions. Total RNA was isolated from transiently transfected cells (TRIzol reagent, Invitrogen, CA), reverse transcribed (Superscript III, Invitrogen, CA), and subjected to quantitative PCR analysis using SYBER green master mix (Invitrogen, CA). The comparative threshold cycle (Ct) method was used to calculate the amplification factor, and the relative amount of targets (*Rpl26* and *2810001G20Rik*) was normalized to *Gapdh* levels in parallel reactions. The primer sequences used for qPCR are as follows: *Rpl26*, 5′-CGA AGC AAG AAC CGC AAA CGG C and 3′-ACC ACC TTG CCA ATC TGC TGG C; *2810001G20Rik*, 5′-TGG GAA TGA ACC CTG GCG CTGA and 3′-TTG GGC ACA GCA TCC GTC TTG G; *Gapdh*, 5′-TTT CCT CGT CCC GTA GAC AAA A and 3′-CGT TGA ATT TGC CGT GAG TGG.

### RNA sequencing analysis

Mice were euthanized for RNA sequencing analysis at 100 days old after 30 days of treatment with SD or PBS. The spinal cord was snap-frozen on dry ice and total RNA was extracted immediately from the lumbar portion using the RNeasy Lipid Tissue Mini-kit (Qiagen Inc., Valencia, CA, USA). RNA was measured in a spectrophotometer at 260 nm absorbance. For the mRNA-Seq sample preparation, the Illumina standard kit was used according to the manufacturer’s protocol. Briefly, 3 µg of each total RNA sample was used for polyA mRNA selection using streptavidin-coated magnetic beads, followed by thermal mRNA fragmentation. The fragmented mRNA was subjected to cDNA synthesis using reverse transcriptase (SuperScript II) and random primers. The cDNA was further converted into double-stranded cDNA and, after an end repair process (Klenow fragment, T4 polynucleotide kinase and T4 polymerase), was finally ligated to Illumina paired end (PE) adaptors. Size selection was performed using a % agarose gel, generating cDNA libraries ranging in size from 200 to 250 bp. Finally, the libraries were enriched using ten cycles of PCR and purified by the QIAquick PCR purification kit (Qiagen). The enriched libraries were diluted with Elution Buffer to a final concentration of 10 nM. Each library was run at a concentration of 8 pM on one Genome Analyzer (GAIIx) lane using 53 bp sequencing. Raw reads were aligned to the mouse genome (GRCm38, mm10) according to the STAR as an accurate alignment tool for high-throughput RNA-seq data [42]. Moreover, according to Ensembl gene set, we used HTSeq to count the reads aligned to each gene [43].

### Western blot analysis

For immunoblot analysis, cells were harvested and washed with ice-cold PBS and then resuspended in an ice-cold cell extraction buffer containing 50 μm Tris-HCl, pH 7.4, 150 μm NaCl, 2 μm EDTA, 1% Triton X-100, 1 μm PMSF, 10 mg/ml leupeptin, 1 μm pepstatin, 1 μm *N*-ethylmaleimide, 2 μm Na_3_VO_4_, 20 μm sodium pyrophosphate, and 50 μm NaF. Lysates were centrifuged at 15,000 rpm at 4 °C for 30 min. The clear cytosol was separated from the insoluble pellet and immediately used for immunoblot. The supernatants were removed carefully and the protein concentration was quantified by Bradford Method (Bio-Rad, Hercules, CA). Lysates were mixed with 2× or 5× boiling Laemmli’s buffer (1× is 100 μm Tris-HCL, pH 6.8, 4% SDS, 200 μm dithiothreitol, 20% glycerol, 2% SDS, 0.2% bromophenol blue, 10 mg/ml aprotinin, 10 mg/ml leupeptin). Then the samples were boiled for 10 min and then spun at 15,000 rpm for 10 s. In general, 30 µg of protein was electrophoresed on 10% SDS-polyacrylamide gel and transferred to nitrocellulose membrane. Membranes were blocked in 5% skim milk in TBST (Tris, pH 7.4; 150 μm NaCl; 0.05% Tween 20) for 30 min at room temperature. Blots were probed with primary antibodies overnight at 4 °C. This was followed by incubation with anti-rabbit or anti-mouse IgG conjugated with horseradish peroxidase (Bio-Rad, Hercules, CA) for 1 h. Signals were detected by using the ECL system (Amersham Corp., Arlington Heights, IL).

### Analysis of H3K4me2 ChIP-sequencing data from the public resource

The mouse H3K4me2 ChIP-seq data was downloaded from GSE123652. Sequence reads were trimmed and aligned to the Mus musculus genome version mm10 using Bowtie2 [44]. Then, we identified the peaks of H3K4me2, or regions of the genome where more reads are present than random using HOMER (Hypergeometric Optimization of Motif EnRichment) [45]. Finally, identified peaks were annotated with the motifs using the annotatePeaks.pl function. Genes with the occupancy of H3K4me2 significantly altered in their promoter region were identified as H3K4me2 target genes.

### Statistics

Survival data was analyzed by the Kaplan–Meier survival curves. Values were analyzed by one-way ANOVA and the Brown–Forsythe and Welch test followed by Kolmogorov–Smirnov multiple-comparison test, unless indicated otherwise. All statistics and graphs were performed using Prism 8 (GraphPad Software, San Diego, CA). *p* < 0.05 was considered significant.

## Results

### LSD1 is induced in a cellular model of ALS and plays a role as a transcriptional repressor

**Fig. 1:**
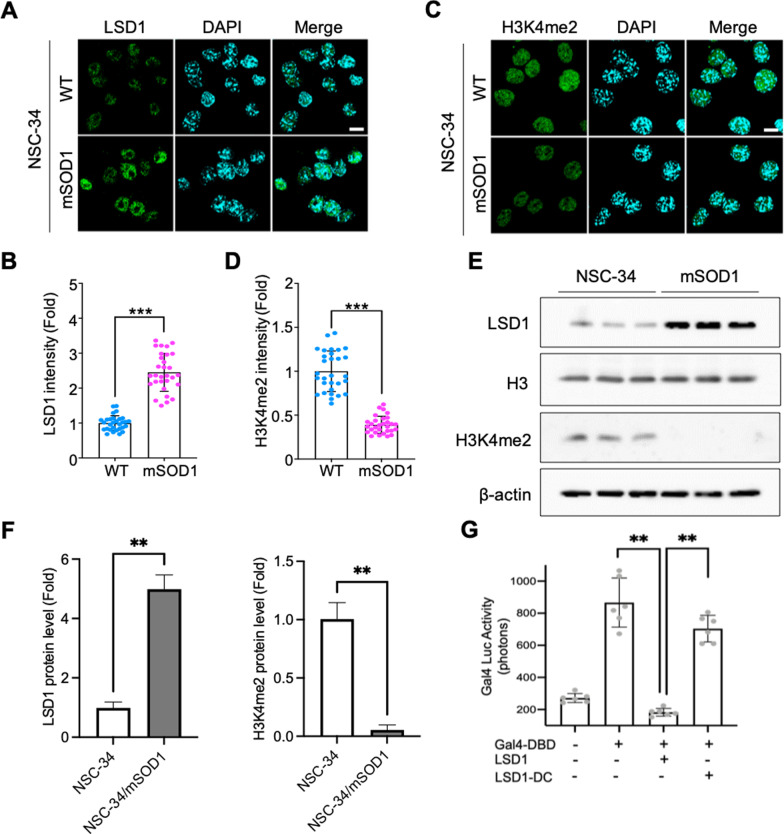
LSD1 is induced in a cellular model of ALS and plays a role as a transcriptional repressor.** A** LSD1 immunoreactivity (green) was increased in the nucleus of mSOD1 (G93A) NSC-34 motor neuron-like cells. The nucleus was counterstained with DAPI (blue) and scale bar is 10 μm (white): **B** Quantification of LSD1 immunoreactivity level. The number of cell counting: 30 cells/group. **C** H3K4me2 immunoreactivity (green) is decreased in NSC-34/mSOD1 cells. **D** Quantification of H3K4me2 immunoreactivity level. The number of cell counting: 30 cells/group. **E** Western blot analysis shows that LSD1 protein level was increased while H3K4me2 protein level was decreased in NSC-34/mSOD1 cells compared to NSC-34 motor neuron cells. **F** Quantification of LSD1 and H3K4me2 protein level. **G** LSD1 significantly repressed Gal4-DBD-driven transcriptional activity in SH-SY5Y neuroblastoma cells. Gal4-DBD, Gal4-LSD1, and Gal4-LSD1-DC (a deletion mutant of histone demethylase c-terminus domain) plasmids were transiently transfected together with Gal4-luciferase reporter gene. The data are the means ± SEM of three independent experiments. Significantly different at *p < 0.05 and **p < 0.01

In order to examine LSD1 expression in a cellular model of ALS, we used spinning disk confocal microscopy imaging and Western blot analysis to detect LSD1 immunoreactivity. Using motor neuronal cell line (NSC-34) as a model, we transfected wild type (WT)-SOD1 and mSOD1 (G93A) overexpression constructs and observed for LSD1 and H3K4me2 expression. Images and densitometry results demonstrate increase of LSD1 immunoreactivity (Fig. 1A and B) and reduction of H3K4me2 immunoreactivity (Fig. 1C and D) in WT-SOD1 compared to mSOD1 cells. Moreover, western blot analysis showed that the level of LSD1 is elevated while the level of H3K4me2 is reduced in NSC-34/m-SOD1 cells in comparison to NSC-34 motor neuron cells (Fig. 1E and F). The increase of LSD1 and reduction of H3K4me2 immunoreactivity can be seen in N2a cell lines (Additional file 1: Fig. S1). Previous studies identified that LSD1 plays an epigenetic modifier role by removing dimethyl groups from the fourth positions on histone 3 protein (H3K4me2) associated with gene repression [25, 26, 46]. To determine whether the alteration of LSD1 level is associated with the transcriptional repression in neurons, we overexpressed LSD1 transiently and measured Gal4-DBD-driven luciferase activity as shown in Fig. 1G. This result shows LSD1 expression suppresses transcription in transiently transfected SH-SY5Y neuroblastoma cells suggesting that LSD1 expression can regulate transcriptional activity in neurons.

### LSD1 is induced by oxidative stress in a motor neuronal cell line (NSC-34)

**Fig. 2:**
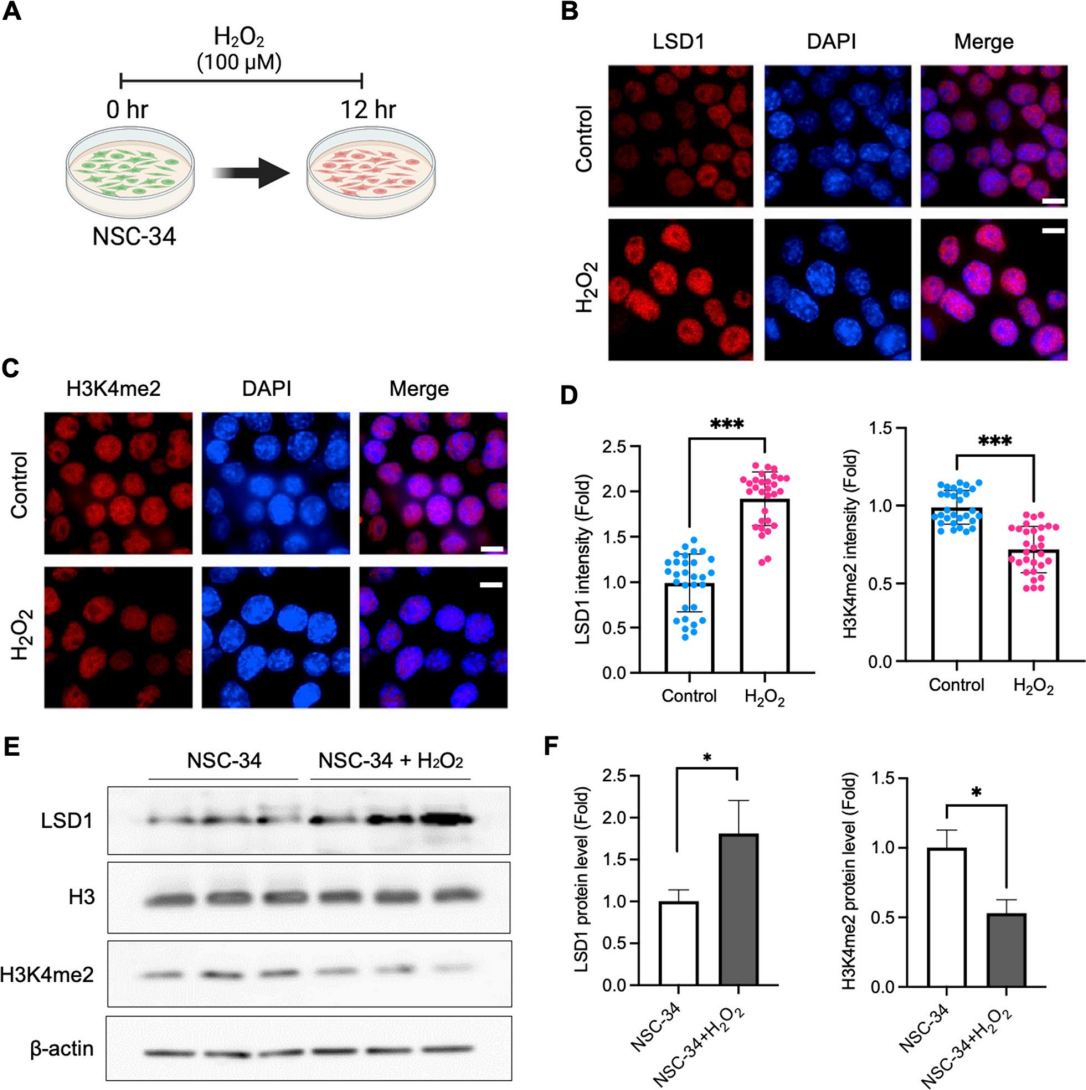
LSD1 is induced by oxidative stress in a motor neuronal cell line (NSC-34).** A** A scheme illustrating a work flow of cell experiment. **B** Confocal microscopy shows that oxidative stress increased LSD1 immunoreactivity (red) in the nucleus of NSC-34 cells. The nucleus was counterstained with DAPI (blue). Scale bars (white): 10 μm. **C** Confocal microscopy shows that oxidative stress (H_2_O_2_) reduced H3K4me2, a substrate of LSD1, in NSC-34 cells. Scale bars (white): 10 μm. **D** Quantification of LSD1 and H3K4me2 immunoreactivity level. The number of cell counting: 30 cells/group. **E** Western blot analysis confirmed that oxidative stress increased LSD1 level but decreased H3K4me2 level in the NSC-34 motor neuron cells. **F** Quantification of LSD1 and H3K4m2 levels from Western blot analysis (originated from **E**). Significantly different at *p < 0.05, **p < 0.01, and ***p < 0.001

Oxidative stress is an important factor in the pathogenesis of most neurodegenerative diseases including ALS [16, 47]. In this context, it is reasonable to investigate how the level and activity of LSD1 and H3K4me3 is associated with the pathogenesis of ALS. To investigate the immunoreactivity of LSD1 and H3K4me2 in response to oxidative stress, we exposed 100 µM hydrogen peroxide (H_2_O_2_) to motor neuronal cells (NSC-34) for 12 h (Fig. 2A). The results demonstrated an increase of LSD1 immunoreactivity (Fig. 2B) and a reduction of H3K4me2 immunoreactivity (Fig. 2C). The densitometry results also show the significant changes of LSD1 and H3k4me2 immunoreactivity in NSC-34 cells exposed by oxidative stress (Fig. 2D). Western blot analysis showed that oxidative stress (1 µM of H_2_O_2_ for 6 h) elevated the level of LSD1 while it reduced the level of H3K4me2 in the NSC34 cells (Fig. 2E). The densitometry analysis exhibited significant changes of LSD1 and H3K4me2 levels (Fig. 2F). Moreover, we verified that deferoxamine (DFO), an iron chelator and antioxidant, decreased LSD1 and elevated the H3K4me2 levels by nullifying hydrogen peroxide (H_2_O_2_)-induced oxidative stress in NSC-34/mSOD1 cells (Additional file 1: Fig. S2). In addition, DFO elevated the level of H3K4me2 in H_2_O_2_-treated NSC-34 motor neuronal cells. But DFO maintained the levels of LSD1 and H3K4m2 similar to the H_2_O_2_-treated NSC-34/WT-SOD1 (Additional file 1: Fig. S2).

### LSD1 knock-down by shLSD1 increases the level of H3K4me2 but not trimethylated H3K4 (H3K4me3)

**Fig. 3:**
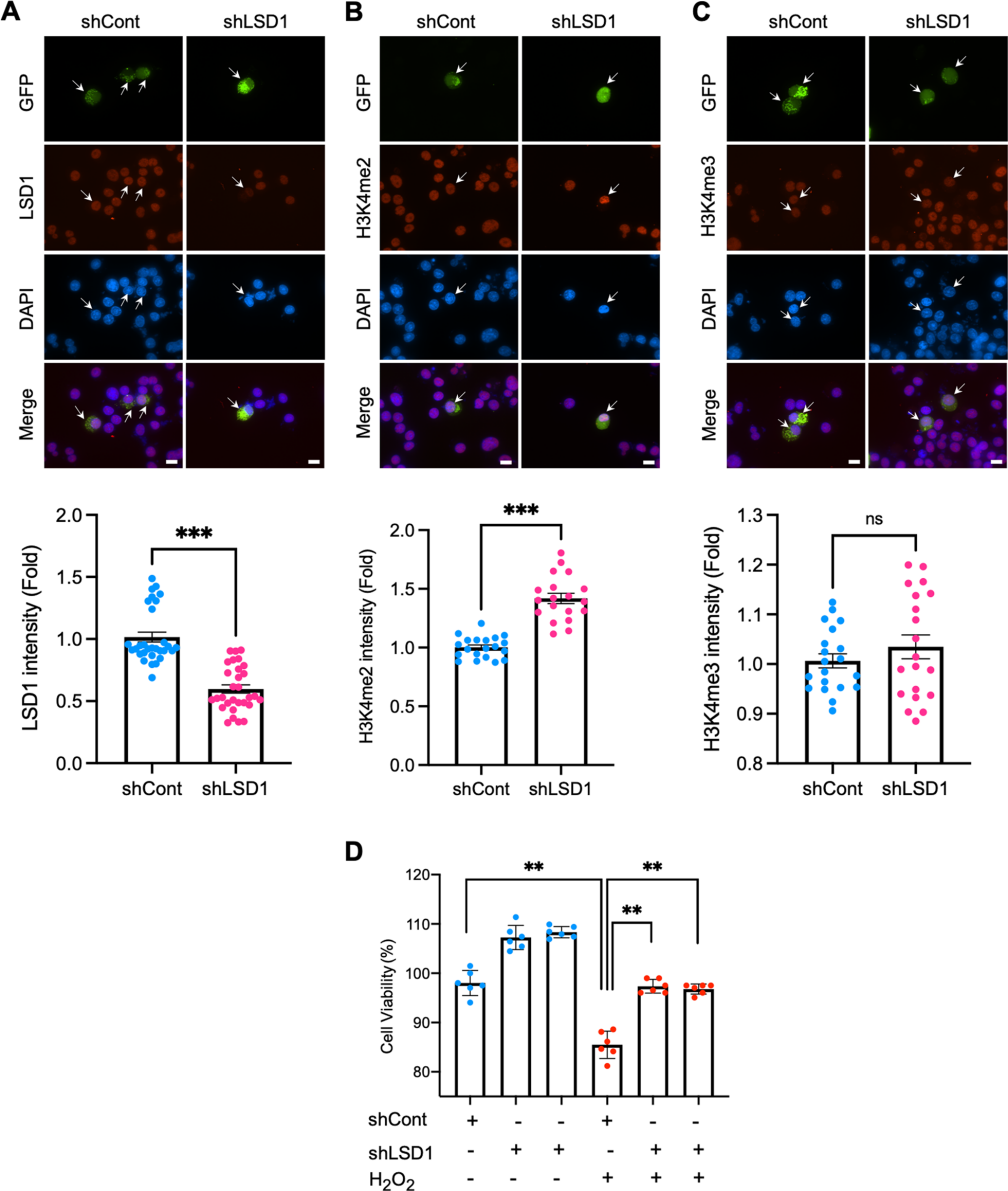
LSD1 knock-down by shLSD1 increases the level of H3K4me2 but not H3K4me3, and ameliorates neuronal survival.** A** shLSD1-GFP but not shControl-GFP reduced endogenous LSD1 levels. Arrows indicate GFP-positive cells expressing shRNA. Scale bars (white): 10 μm. The densitometry analysis shows that LSD1 is significantly reduced by shLSD1-GFP (bottom panel). The number of cells counting: 30 cells/group. **B** shLSD1-GFP decreased H3K4me2 immunoreactivity. The densitometry analysis shows that H3K4me2 is significantly reduced by shLSD1-GFP (bottom panel). The number of cells counting: 20 cells/group. **C** H3K4me3 immunoreactivity was not altered by shLSD1-GFP expression. Densitometry analysis shows that H3K4me3 was slightly increased by shLSD1-GFP but not significant (bottom panel). The number of cells counting: 20 cells/group. **D** Knock-down of LSD1 (shLSD1) improved neuronal survival under oxidative stress conditions. shRNA control (shCont) and shLSD1 (500 ng/ml) were transiently transfected in N2a cells for 36 h. Cells were exposed to 100 µM of H_2_O_2_ for 12 h and viability was measured by MTT assay. Data was derived from three independent experiments (duplicates in each experiment). Significantly different at *p < 0.05, **p < 0.01, and ***p < 0.001

To verify whether LSD1 is involved in the demethylation of H3K4me2, we utilized RNAi technology to knock-down endogenous LSD1 in N2a cells and measured for immunoreactivity of LSD1, H3K4me2, and H3K4me3 using spinning disk confocal microscopy (Fig. 3). mSOD1 (G85R) N2a cells were transiently transfected with either shControl- or shLSD1-GFP plasmids for 48 h and immunoreactivity of LSD1, H3K4me2, and H3K4me3 was imaged. Unlike shControl-GFP, shLSD1-GFP reduced endogenous LSD1 expression levels (Fig. 3A). In these images, GFP-positive cells represent the expression of shRNA plasmids. Based on the results, H3K4me2 immunoreactivity is increased by shLSD1-GFP expression (Fig. 3B), while H3K4me3 immunoreactivity is not altered by shLSD1-GFP expression (Fig. 3C). Furthermore, Fig. 3D shows improvement in neural survival under oxidative stress conditions by LSD1 (shRNA) knock-down.

### LSD1 is modulated in the motor neurons of an animal model of ALS

**Fig. 4:**
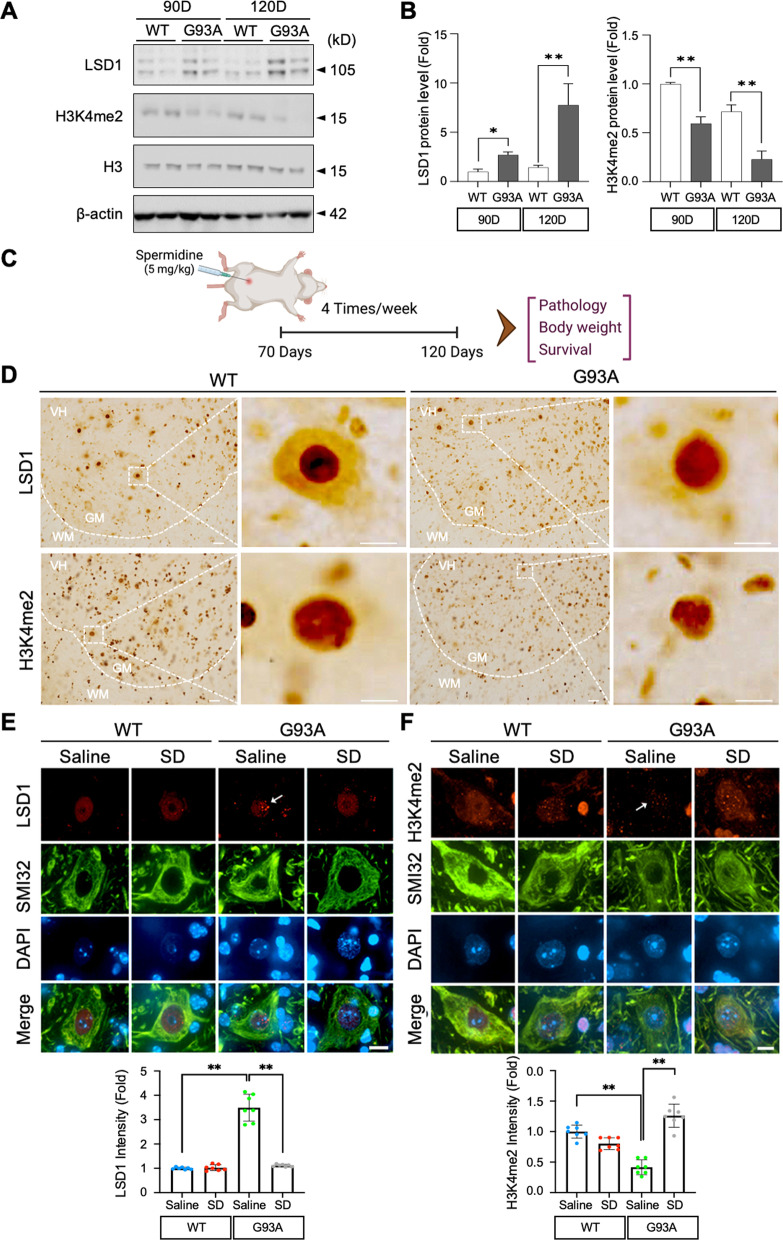
LSD1 is elevated in the motor neurons of an animal model of ALS.** A** Western blot analysis of LSD1 expression assessment in ALS (G93A) mice compared to WT at 90 and 120 days of age. **B** The densitometry analysis shows a significant increase of LSD1 and decrease of H3K4me2 protein level in G93A mice. **C** A scheme illustrating a work flow of animal experiments. **D** Immunoreactivity of LSD1 (brown) and H3K4me2 was mainly found in the nucleus of motor neurons in ventral horn of WT and G93A mice. **E** SD administration decreased the punctate structures of LSD1 immunoreactivity in the nucleus of motor neurons of ALS (G93A) mice. The densitometry analysis shows that LSD1 is significantly reduced by SD (bottom panel). The number of cell counting: 7 cells/group. **F** SD administration restored the H3K4me2 immunoreactivity in the nucleus of motor neurons of ALS (G93A) mice. An arrow indicates a motor neuron with the lowered level of H3K4me2. The densitometry analysis shows that H3K4me2 is significantly elevated by SD (bottom panel). Scale bars (white): 10 μm. The number of cell counting: 7 cells/group. Significantly different at *p < 0.05 and **p < 0.01

To further determine whether LSD1 is regulated in an animal model of ALS, we compared LSD1 immunoreactivity in the motor neurons of mSOD1 (G93A) mouse to that of WT mouse. Using spinning disk confocal microscopy, we examined SMI32-positive motor neuron sections of the lumbar spinal cord. The Western blot results indicate increasing LSD1 and decreasing H3K4me2 protein level at both 90 and 120 days of age in ALS (G93A) mice compared to WT (Fig. 4A and B). Previous studies verified that intraperitoneal injection of SD crosses blood-brain barrier and affects central nervous system-associated functions in mice [48–51]. Accordingly, in order to examine the role of SD in ALS (G93A) mice, we performed intraperitoneal injection (I.P. injection) of SD from 70 to 120 days of age followed by neuropathological, body weight and survival evaluation (Fig. 4C). The immunohistochemistry images showed the immunoreactivity of LSD1 was mainly found in the nucleus of motor neurons in the ventral horn of WT and G93A mice (Fig. 4D). Moreover, the immunofluorescence staining image of single motor neuron showed that LSD1 immunoreactivity was diffusely localized in the nucleus of WT mouse sections, while the number of LSD1 punctate structures was two-fold more in mSOD1 (G93A) mice than WT mice. The sections from SD treated mSOD1 mouse showed a reduction in LSD1 punctate structures (Fig. 4E). On the other hand, the immunoreactivity levels of H3K4me2 decreased in the motor neurons of the ALS mouse model, while H3K4me2 immunoreactivity was diffusely localized in the nucleus of SMI32-positive motor neurons in lumbar spinal cord of WT mouse (Fig. 4F). It can also be seen that SD treatment restores the immunoreactivity of H3K4me2 in the nucleus of motor neurons of ALS (G93A) mouse.

### SD protects motor neurons and modulates LSD1 activity in the lumbar spinal cord in ALS (G93A) mice

**Fig. 5:**
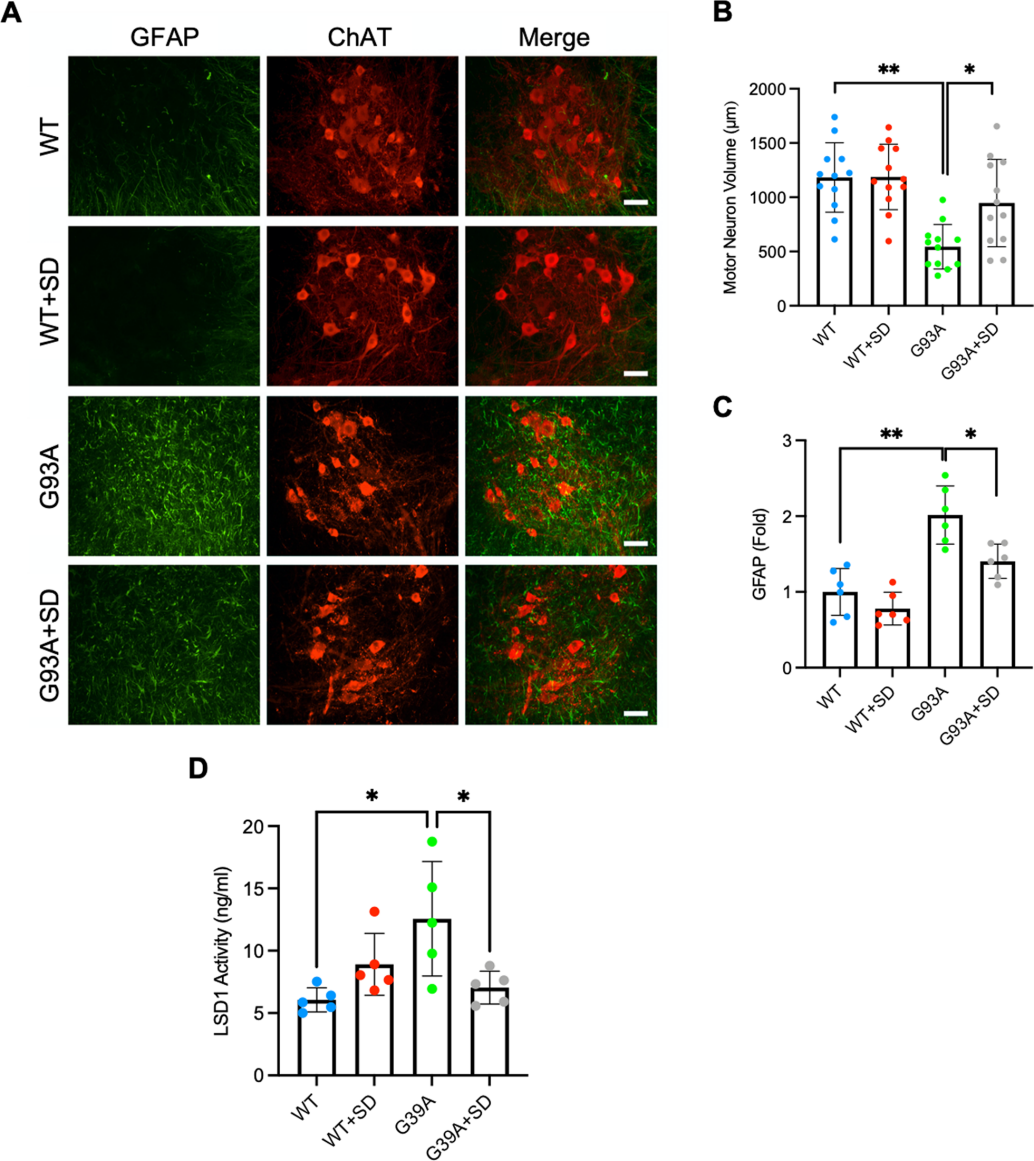
SD administration prevents loss of motor neurons and modulates LSD1 activity in the lumbar spinal cord of G93A mice.** A** SD administration prevented loss of ChAT-positive motor neurons (red) while reducing the immunoreactivity of GFAP (green) in the ventral horn of G93A mice. Scale bars (white): 50 μm. **B** SD administration improved the volume (µm^3^) of the motor neuron cell body. The number of cell counting: 12 cells/group. **C** SD significantly decreased the immunoreactivity of GFAP in G93A mice compared to WT mice. The intensity of GFAP immunoreactivity (in six foci of the ventral gray matter) was analyzed by NIH ImageJ program. **D** LSD1 activity was significantly elevated in mSOD1 ALS (G93A) mice (n = 5) in comparison to WT mice (n = 5). SD administration decreased the LSD1 activity in mSOD1 ALS (G93A) mice (n = 5) but not in WT mice (n = 5) at 120 days of age. SD and saline were separately administered (5 mg/kg, I.P. injection) to groups of 6 wildtype and G93A mice from 30 to 120 days of age. Significantly different at *p < 0.05 and **p < 0.01

The results show the marked motor neuron loss, atrophy, and increased astrogliosis in the lumbar spinal cord in control G93A mice (Fig. 5A), whereas the ALS (G93A) mice receiving SD treatment clearly preserve motor-neuron population compared to their control counterparts (Fig. 5A). To measure size change in motor neurons, we analyzed cell body volume using spinning confocal microscopy with AQI-X-COMBO-CWF image analysis program (Media cybernetics Inc. Bethesda, MD). The volume of choline acetyltransferase (ChAT)-positive motor neurons was significantly reduced in the lumbar spinal cord tissue of mSOD1 (G93A) mice compared to littermate control mice. SD treatment prevented atrophy of motor neurons in ALS (G93A) mice (Fig. 5B). On the other hand, several previous studies have proven the non-cell autonomous pathway in which reactive astrocytes contribute to motor neuronal damage in ALS [52, 53]. Now, it is well-accepted that astrocytes are determinants of disease progression in ALS. In this context, we examined how SD affects the reactivity of astrocytes in ALS (G93A) mice. Interestingly, our results showed that SD reduced the immunoreactivity of GFAP, indicating that SD prevented the activation of astrocytes in mSOD1 ALS (G93A) mice (Fig. 5C). Furthermore, LSD1 activity, which increased in mSOD1 ALS (G93A) mice, decreased in ALS mice injected by SD at 120 days of age (Fig. 5D).

### SD improves motor activity and gait in ALS (G93A) mice

**Fig. 6:**
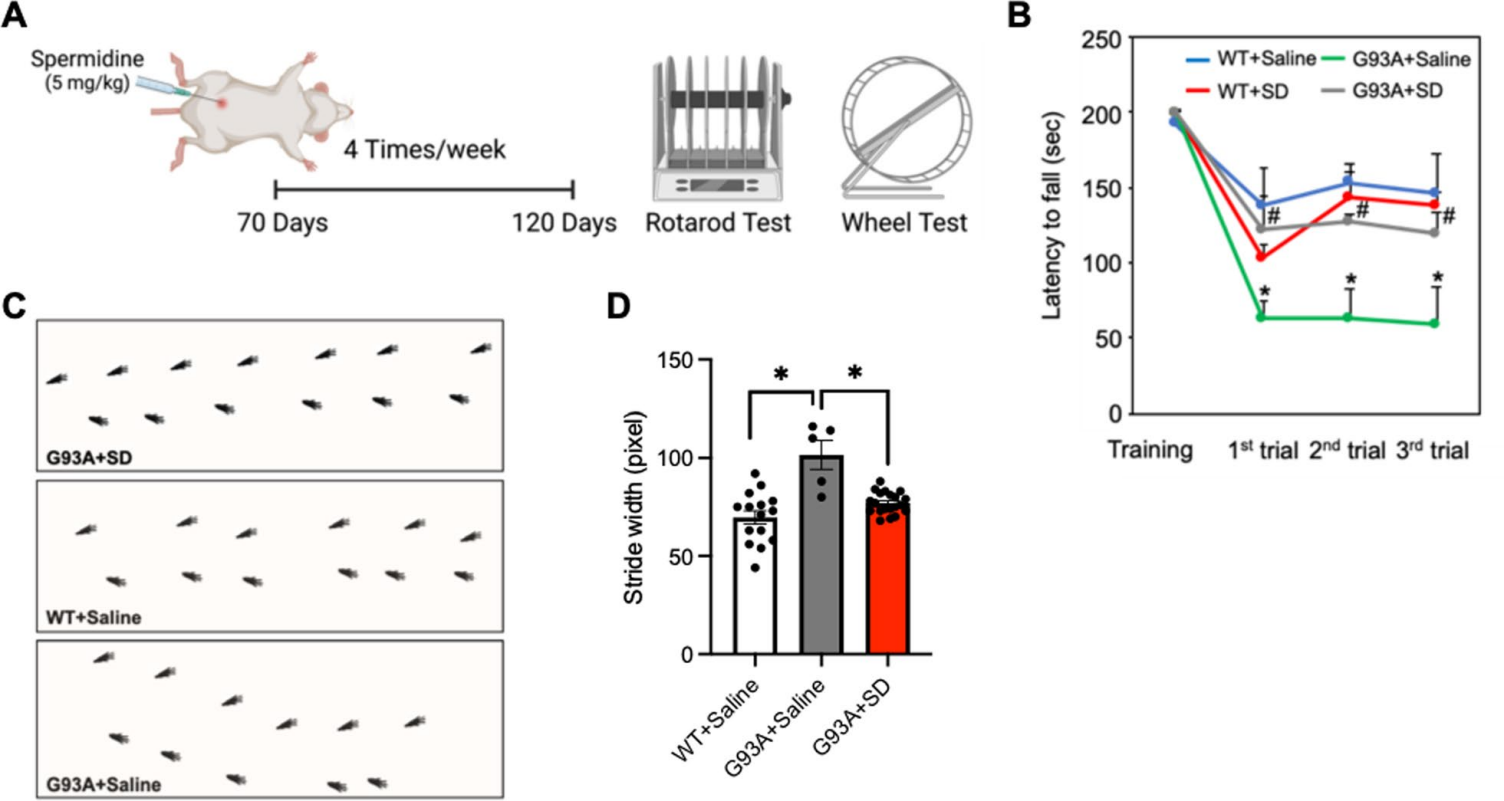
SD administration improves rotarod and gait performance of ALS (G93A) mice.** A** SD was administered to the mice from 70 to 120 days of age, then rotarod and wheel tests were performed. **B** SD administration significantly improved rotarod performance of ALS (G93A) mice (n = 3) compared to vehicle (saline)-treated ALS mice (n = 4). **C** Footprint analysis from the accelerating wheel test. **D** Gait analysis showed that SD administration improved the increase of stride width in ALS mice. Significantly different at *p < 0.05

SD was administered to mice 5 times a week from 70 to 120 days of age. Then accelerating rotarod and wheel tests were performed to evaluate the motor function of the mice (Fig. 6A). The SD-treated ALS (G93A) mice exhibited significantly improved rotarod performance compared to vehicle-treated ALS mice (Fig. 6B). In the gait analysis, ALS (G93A) mice showed the wider stride width than WT (Fig. 6C) and the stride width was recovered in SD-treated ALS (G93A) mice (Fig. 6D).

### SD delays onset of disease and prolongs the life span of ALS (G93A) mice

**Fig. 7:**
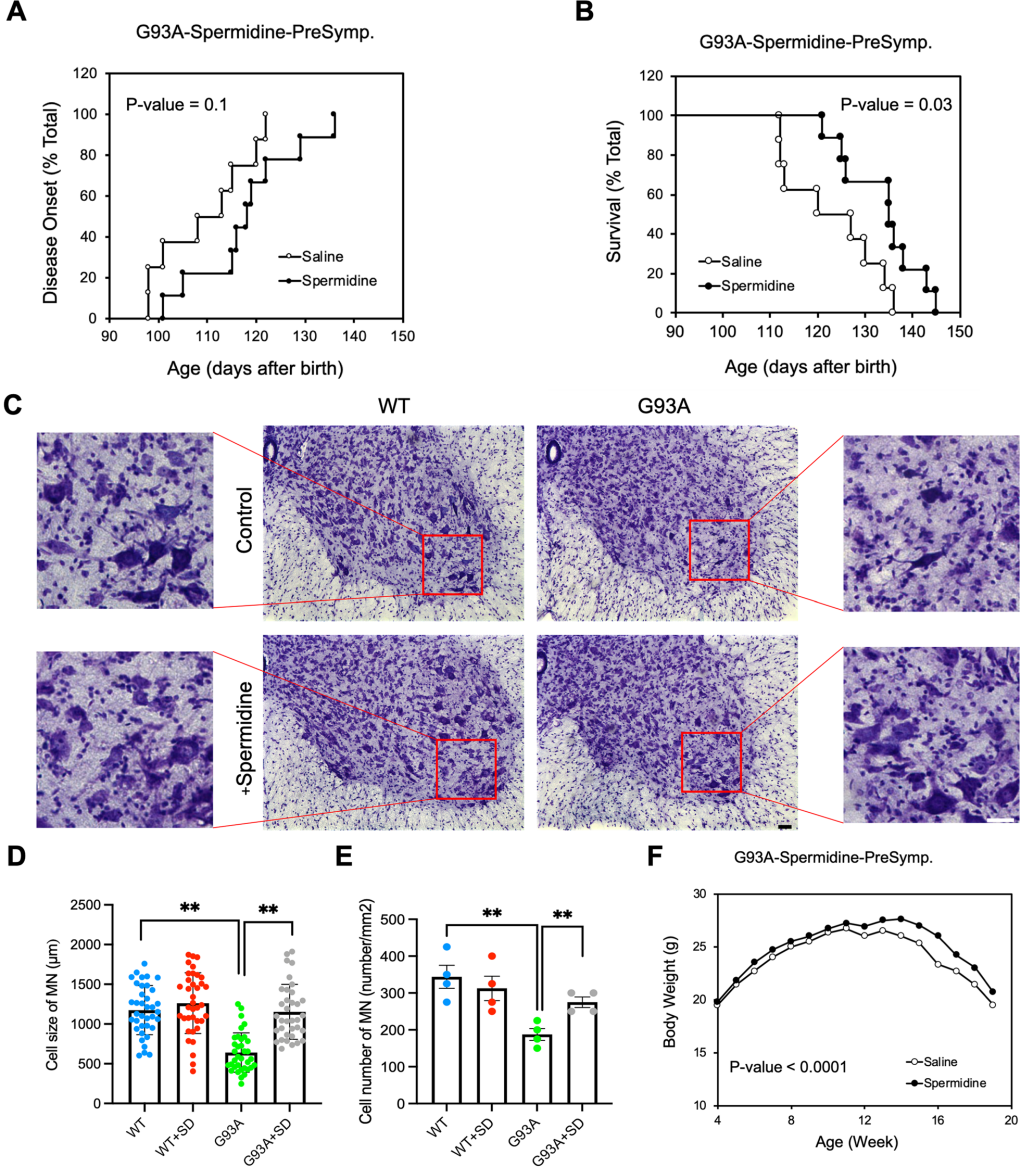
SD prolongs the life-span of ALS (G93A) mice.** A** Pre-symptomatic supplementation of SD delayed disease onset in ALS mice (n = 10) compared to vehicle (saline) control (n = 10). **B** SD administration prolonged the life-span of ALS mice as shown by Kaplan–Meier probability of survival analysis. The statistics were calculated by Mantel–Cox test. **C** SD administration prevented loss of motor neurons in the ventral horn of spinal cord in G93A mice. Lumbar spinal cord tissue sections were stained with cresyl violet (Nissl staining). Scale bars: 20 μm. **D** SD administration improved the size of motor neurons. The number of cell counting: 40 cells/group. **E** SD administration restored a total number of motor neurons in the ventral horn of G93A. **F** SD administration improved the bodyweight of ALS mice (n = 10) compared to vehicle (saline)-treated ALS mice (n = 10) after 14 weeks. The statistics for body weight was calculated by Wilcoxon matched-pairs signed rank test. Significantly different at *p < 0.05 and **p < 0.01

Consistent with neuropathology results, SD treatment leads to a delay in disease onset, extended survival, and the ability to maintain the body weight of ALS mice. Figure 7A demonstrates that SD significantly delayed onset of disease. Furthermore, SD significantly extended survival in pre-symptomatic G93A mice by 20% compared to untreated mSOD1 (G93A) mice (Fig. 7B). Kaplan-Meier probability of survival analysis show that pre-symptomatic supplementation of SD more effectively prolonged the survival of ALS mice. The marked reduction of ventral neuronal loss by SD can be seen in Nissl-stained tissue sections from the lumbar spinal cord of G93A mice (Fig. 7C–E). Furthermore, SD significantly improved body weight in pre-symptomatic mSOD1 (G93A) mice compared to untreated mSOD1 (ALS) mice (Fig. 7F).

### Functional analysis of DEGs in ALS (G93A) mice and G93A mice treated by SD

**Fig. 8:**
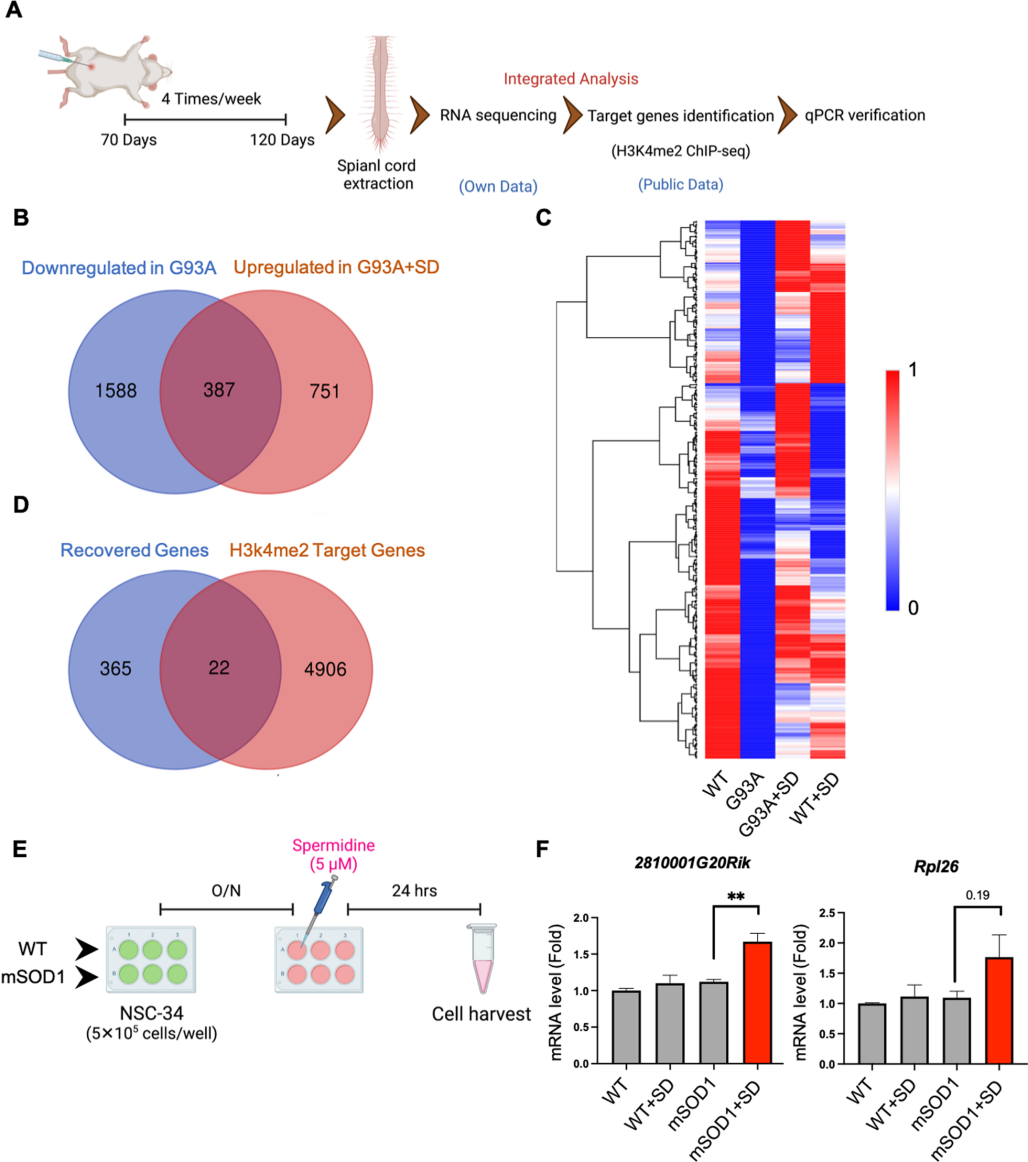
Functional analysis shows that SD modulates gene expression in ALS transgenic (G93A) mice and motor neuron cell line (NSC-34).** A** A scheme illustrating procedure of data analysis. **B** Venn diagram of the total number of downregulated genes in G93A mice that upregulated in G93A mice treated by spermidine (G93A + SD). **C** Heatmap analysis shows that SD modulates the expression level of 387 genes in G93A mice. **D** Venn diagram of 387 recovered genes by SD versus H3K4me2 ChIP target genes. **E** A scheme illustrating a work flow of cell experiment. **F** *2810001G20Rik* and *Rpl26* gene expressions are upregulated by SD in NSC-34/mSOD1cells compared to wildtype control cells. The graph represents mean-SEM of three separate experiments. Significantly different at **p < 0.01

To find out target genes regulated by SD, first, we performed RNA-seq on the lumbar spinal cord extracted from four kinds of mice such as WT mice, G93A ALS mice, WT mice + SD (spermidine administration) and G93A ALS mice + SD (GSE213091). After we identified SD-modulated targets from the spinal cord RNA-seq, we retrieved the public H3K4me2 ChIP-seq (GSE123652) and analyzed whether H3K4me2-landscaped genes are associated with our RNA-seq data. Lastly, H3K4me2-associated target genes were verified by qPCR on NSC-34 motor neuronal cells (Fig. 8A). Consistent with the known function of LSD1 as a transcriptional repressor, and increase of LSD1 immunoreactivity in ALS (G93A) mice model, differential gene expression analysis identified 1975 downregulated genes in G93A mice with 1.5-fold change (Additional file 2: Table S1). Among those, 387 genes were recovered in G93A mice treated by SD (Fig. 8B). The heatmap also shows the recovered genes in G93A mice treated by SD (Fig. 8C). Among those recovered genes, 22 genes can be targeted by H3K4me2 based on the prefrontal cortex H3K4me2 ChIP-seq data (GSE123652) (Fig. 8D) [54]. Recent study shows the decrease of nuclei count, neuron count and the total neurite length in *2810001G20Rik* knock-down on embryonic cell derived neurons [55]. Moreover, substantial evidence demonstrates 60S ribosomal subunits as a major feature of the tau interactome [56], whereas another study shows total tau levels, which inhibits general ribosomal function, are increased in ALS patients with *C9orf72* mutation [57]. Therefore, *2810001G20Rik* and *Rpl26* genes were considered as two target genes which are downregulated in ALS mice model and recovered by SD. We treated NSC-34 cells by 5 µM of SD and performed quantitative PCR to check the expression levels of *2810001G20Rik* and *Rpl26* on both NSC-34/WT-SOD1 and NSC-34/mSOD1 cells treated by SD (Fig. 8E). The results showed significant increase of mRNA levels of both genes in NSC-34/mSOD1 cells treated by SD compared to cells that were not (Fig. 8F).

## Discussion

### Epigenetic histone modifications in ALS

DNA methylation, histone modification, and microRNAs are the major epigenetic mechanisms in the etiology of ALS. Histones are substrates for a number of covalent modifications that control chromatin state and gene expression. The core histones (H2A, H2B, H3, and H4) are small basic proteins that form nucleosomes or the fundamental building blocks of chromatin structure [58]. Their function is to compress over 2 m of genomic DNA into a nucleus with a diameter of 10 microns. Indeed, histone modifications that include phosphorylation, acetylation, and methylation, play an important role in gene regulation [59, 60]. It has recently been shown that LSD1 is a nuclear amine oxidase homolog that demethylates histone H3 lysine 4, which is correlated with active transcription [61–63]. Shi et al. have described the discovery of LSD1 as an enzyme that has remained elusive for decades and whose existence has been questioned [61]. This protein specifically acts on H3K4me2, and the LSD1-catalysed reaction regenerates the methyl-free lysine together with release of formaldehyde [61, 64]. LSD1 comprises of an N-terminal SWIRM domain and a C-terminal flavin domain which shows homology to the members of the amine oxidase family. These enzymes catalyze the oxidative deamination of compounds that contain primary, secondary, or tertiary amines. LSD1 has been typically found in association with CoREST and histone deacetylases 1 and 2 [37, 64, 65].

Recently, researchers work on dosage-sensitive genes which gain and loss of the same gene involve in neurodevelopmental disorders [66]. Christopher et al. report that the loss of LSD1 leads to paralysis and learning and memory defects along with cortical and hippocampal neurodegeneration in mice [67]. A recent study indicates that the gain or loss of the same gene function is involved in neurodevelopmental disorders in a dosage-dependent manner [66]. Our finding showed that oxidative stress induces motor neuronal damage via, in part, LSD1-H3K4me2 pathway in cellular and mouse model of ALS. Considering previous findings and our data, we propose that either the gain or loss of LSD1, as a dosage-sensitive gene, may contribute to ALS-like pathology and behavioral features [66, 67].

The potential pharmacological value of LSD1 as a drug target stems from it being a flavin-dependent demethylase, rather than an iron-dependent enzyme of the Jumonji class [68]. Therefore, an LSD1 inhibitor is expected to specifically interfere with a single, well-defined demethylation reaction without spurious inhibition of other demethylases. Notably, biguanide and bisguanidine polyamine analogues inhibit LSD1 and are capable of reactivating genes that are pathologically silenced in the disease model [69, 70]. Interestingly, LSD1 shares considerable homology with FAD-dependent polyamine oxidases, including SMO/PAOh1 [37, 71].

### Polyamine therapy

SD, a natural polyamine presented widely in mammalian living cells, has crucial roles in various cellular processes, and extends the lifespan of several model organisms by inducing autophagy [72]. However, the effect of SD against neuronal damage has not yet been clearly determined. Yang et al. suggested inhibition of caspase 3-mediated Beclin 1 cleavage and restoration of the Beclin 1-dependent autophagy as a putative neuroprotective effect of SD [48]. In our previous study, we reported that arginine and polyamine metabolism are deregulated in an animal model of ALS [22]. Based on our current finding, we suggest that the elevation of LSD1 activity and reduction of H3K4me2 level due to impaired polyamine metabolism may lead to transcriptional deregulation of motor neuronal genes. Then, the silencing of survival genes may lead to motor neuronal damage. Additionally, we observe that SD reduced the number of GFAP-positive reactive astrocytes, indicating that it has an effect on the regulation of astrogliosis in ALS. However, we could not precisely determine whether the effects of SD on the epigenetic modifications in the spinal cord is specific or systemic in this study. Accordingly, the specificity of SD effects in the spinal cord remains to be determined in future studies.

**Fig. 9:**
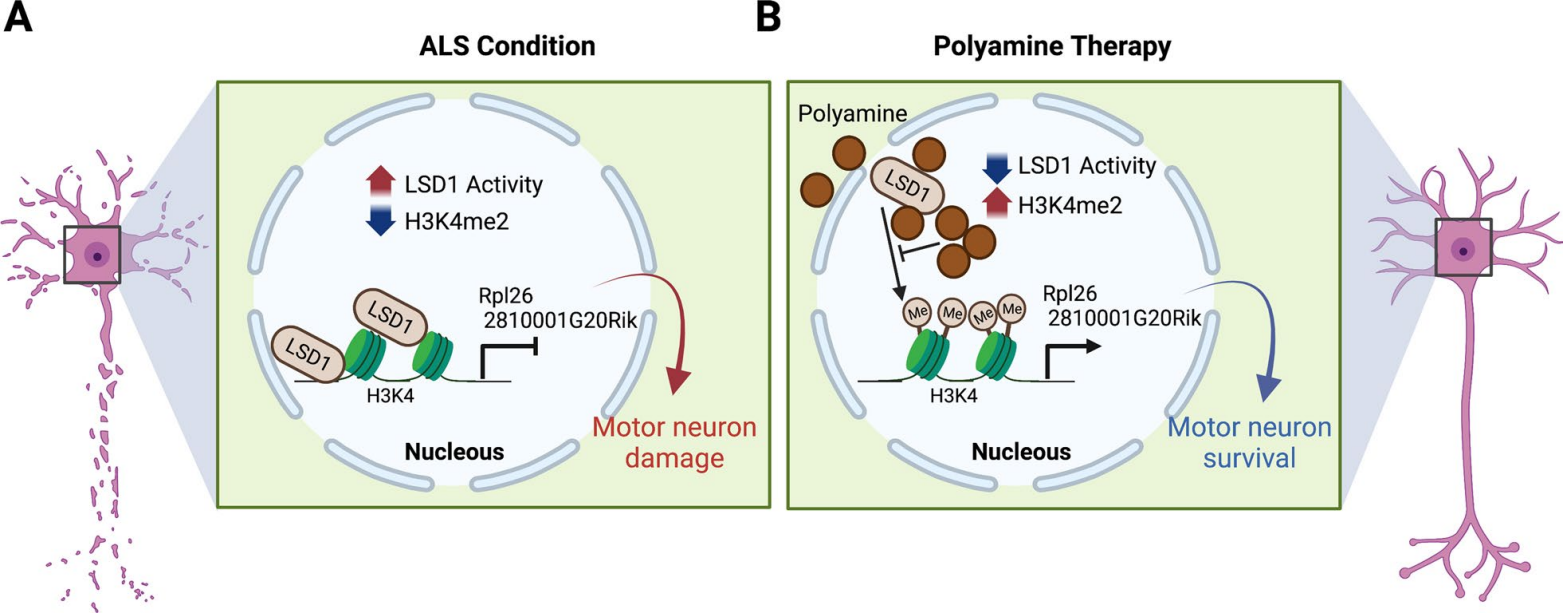
Summarized diagrams depicting the protective role of polyamine (spermidine) against LSD1-H3K4me2 pathway-dependent motor neuronal dysfunction in ALS.** A** In the ALS condition, elevation of LSD1 activity reduces the level of H3K4me2 and subsequently downregulates motor neuronal genes, leading to motor neuronal dysfunction. **B** SD therapy modulates the activity of LSD1 and the expression of epigenomes, ameliorating motor neuron function and neuropathology in ALS. BioRender software has been used to draw the above scheme

In summary, the alteration of LSD1 activity causes a reduction of histone H3K4me2 levels in the ALS mouse model. Our data indicates that polyamines are a negative regulator for LSD1 activity. Indeed, polyamine administration modulated chromatin remodeling and gene expression via the LSD1-H3K4me2-dependent pathway in ALS. Therefore, SD improved neuropathology and extended the survival of ALS mice (Fig. 9). Together, polyamine is an epigenetic modulator and provides a salubrious effect on motor neuronal damage in ALS. Our transcriptome and public ChIP-seq data shows *Rpl26* and *2810001G20Rik* as the candidate genes targeted by H3K4me2 and recovered by SD. Even though the cell line model of ALS did not exactly mimic gene changes in mouse model of ALS, SD treatment affected the expression of motor neuronal genes in both ALS models. Researchers have identified 60 S ribosomal subunits as a major feature of the tau interactome [56]. Moreover, recent study showed that total tau levels, which inhibits general ribosomal function, are increased in ALS patients with *C9orf72* mutation [57]. On the other hand, *Hezroni et al.* demonstrated the decrease of nuclei count, neuron count and the total neurite length in *2810001G20Rik* knock-down on embryonic cell derived neurons [55]. We show that SD treatment improved *2810001G20Rik* and *Rpl26* expression level in a cell line model of ALS.

## Conclusion

ALS is a neurodegenerative disease characterized by progressive paralysis due to motor neuron degeneration. It has been known that epigenetic modifications and its dysfunction contribute to motor neuron death. In this study, we focused on therapeutic approaches to target LSD1 by polyamines and elucidated the mechanistic role of LSD1-H3K4me2-dependent pathway in ALS pathogenesis. Our data indicates that the alteration of LSD1 activity leads to a reduction of histone H3K4me2 levels in the cell line and the mouse model of ALS. Based on our data, we expect that antioxidant may also ameliorate LSD1-H3K4me2 pathway in ALS mice. The effect of antioxidant on epigenetic modulation of ALS pathology and symptoms remains to be examined in the future study. SD (spermidine) administration modulated chromatin remodeling and gene expression via the LSD1-H3K4me2-dependent pathway in ALS. SD, as a negative regulator for LSD1 activity, ameliorated neuropathology and extended the survival of ALS mice. Together, modulating epigenetic targets by small compounds may be a useful therapeutic strategy for treating ALS.

## Supplementary Information

**Additional file 1: Fig. S1.** LSD1 is induced in a cellular model of ALS (N2a cell line). A, LSD1 immunoreactivity (red) was increased in the nucleus of mSOD1 (G85R) N2a cell line. The nucleus was counterstained with DAPI (blue). B, The densitometry analysis shows that LSD1 was significantly increased in G85R N2a cells. The number of cell counting: 35 cells/group. C, Western blot analysis shows that LSD1 protein level was highly induced in mSOD1 (G85R) N2a cell line compared to normal and WT-SOD1 overexpression cell lines. D, Densitometry analysis indicated a significant increase of LSD1 protein level in mSOD1 (G85R) N2a cells. Significantly different at *p < 0.05, ** p < 0.01, and ***p < 0.001. **Fig. S2.** Antioxidant modulates oxidative stress-induced LSD1-H3K4me2 pathway in a cell line model of ALS. A, A scheme illustrating procedure of cell experiment. B, Western blot analysis showed decrease of LSD1 and elevation of H3K4me2 by antioxidant (deferoxamine: DFO) in hydrogen peroxide (H_2_O_2_)-treated NSC-34/mSDO1 cells. C, Densitometry analysis of LSD1 and H3K4m2 levels from Western blot analysis (originated from B). D, Western blot analysis showed the LSD1 and H3K4me2 level maintained by DFO in H_2_O_2_-treated NSC-34/WT-SDO1 cells. E, Densitometry analysis of LSD1 and H3K4m2 levels from Western blot analysis (originated from D). F, Western blot analysis confirmed that DFO elevated the level of H3K4me2 in H_2_O_2_-treated NSC-34 motor neuronal cells.G, Densitometry analysis of LSD1 and H3K4m2 levels from Western blot analysis (originated from F). LSD1 and H3K4me2 were normalized to actin and histone H3 (H3), respectively. Significantly different at *p < 0.05 and **p < 0.01.

**Additional file 2: Table S1.** Integrated analysis of RNA-sequencing and H3K4me2 ChIP-sequencing to identify spermidine-modulated gene signatures in ALS (G93A).

## Data Availability

The RNA-seq data have been submitted to the GEO database (Accession no. GSE213091). All other data is available in the main text or the additional files upon any reasonable requests.

## Abbreviations

ALS: Amyotrophic lateral sclerosis
sALS: Sporadic ALS
fALS: Familial ALS
LSD1: Lysine-specific histone demethylase-1
MAO: Monoamine oxidase
PAO: Polyamine oxidase
FAD: Flavin adenine dinucleotide
SD: Spermidine
I.P. injection: Intraperitoneal injection
ChAT: Choline acetyltransferase
GFAP: Glial fibrillary antigen protein
