# Supplementary material for: Modulation of histone H3K4 dimethylation by spermidine ameliorates motor neuron survival and neuropathology in a mouse model of ALS
Source: J Biomed Sci. 2022 Dec 20;29:106. doi: 10.1186/s12929-022-00890-3 (PMC9764677; doi:10.1186/s12929-022-00890-3)
Supplement: Supplementary file 1 — Additional file 1: Fig. S1. LSD1 is induced in a cellular model of ALS (N2a cell line). A, LSD1 immunoreactivity (red) was increased in the nucleus of mSOD1 (G85R) N2a cell line. The nucleus was counterstained with DAPI (blue). B, The densitometry analysis shows that LSD1 was significantly increased in G85R N2a cells. The number of cell counting: 35 cells/group. C, Western blot analysis shows that LSD1 protein level was highly induced in mSOD1 (G85R) N2a cell line compared to normal and WT-SOD1 overexpression cell lines. D, Densitometry analysis indicated a significant increase of LSD1 protein level in mSOD1 (G85R) N2a cells. Significantly different at *p < 0.05, ** p < 0.01, and ***p < 0.001. Fig. S2. Antioxidant modulates oxidative stress-induced LSD1-H3K4me2 pathway in a cell line model of ALS. A, A scheme illustrating procedure of cell experiment. B, Western blot analysis showed decrease of LSD1 and elevation of H3K4me2 by antioxidant (deferoxamine: DFO) in hydrogen peroxide (H2O2)-treated NSC-34/mSDO1 cells. C, Densitometry analysis of LSD1 and H3K4m2 levels from Western blot analysis (originated from B). D, Western blot analysis showed the LSD1 and H3K4me2 level maintained by DFO in H2O2-treated NSC-34/WT-SDO1 cells. E, Densitometry analysis of LSD1 and H3K4m2 levels from Western blot analysis (originated from D). F, Western blot analysis confirmed that DFO elevated the level of H3K4me2 in H2O2-treated NSC-34 motor neuronal cells.G, Densitometry analysis of LSD1 and H3K4m2 levels from Western blot analysis (originated from F). LSD1 and H3K4me2 were normalized to actin and histone H3 (H3), respectively. Significantly different at *p < 0.05 and **p < 0.01. [file 12929_2022_890_MOESM1_ESM.docx]

**
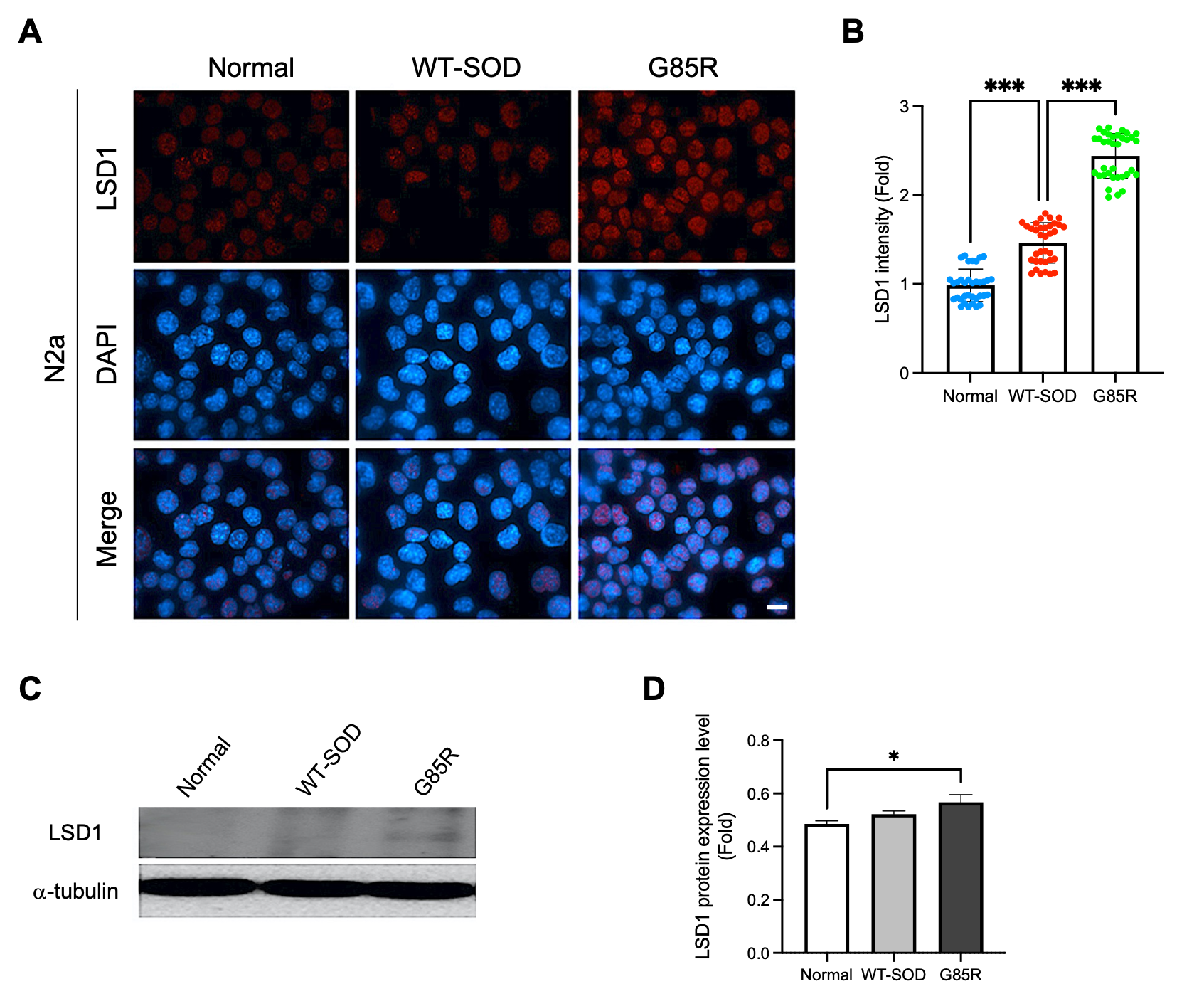
**

**Figure S1. LSD1 is induced in a cellular model of ALS (N2a cell line).** **A**, LSD1 immunoreactivity (red) was increased in the nucleus of mSOD1 (G85R) N2a cell line. The nucleus was counterstained with DAPI (blue). **B**, The densitometry analysis shows that LSD1 was significantly increased in G85R N2a cells. The number of cell counting: 35 cells/group. **C**, Western blot analysis shows that LSD1 protein level was highly induced in mSOD1 (G85R) N2a cell line compared to normal and WT-SOD1 overexpression cell lines. **D**, Densitometry analysis indicated a significant increase of LSD1 protein level in mSOD1 (G85R) N2a cells. Significantly different at *p<0.05, ** p<0.01, and ***p<0.001.


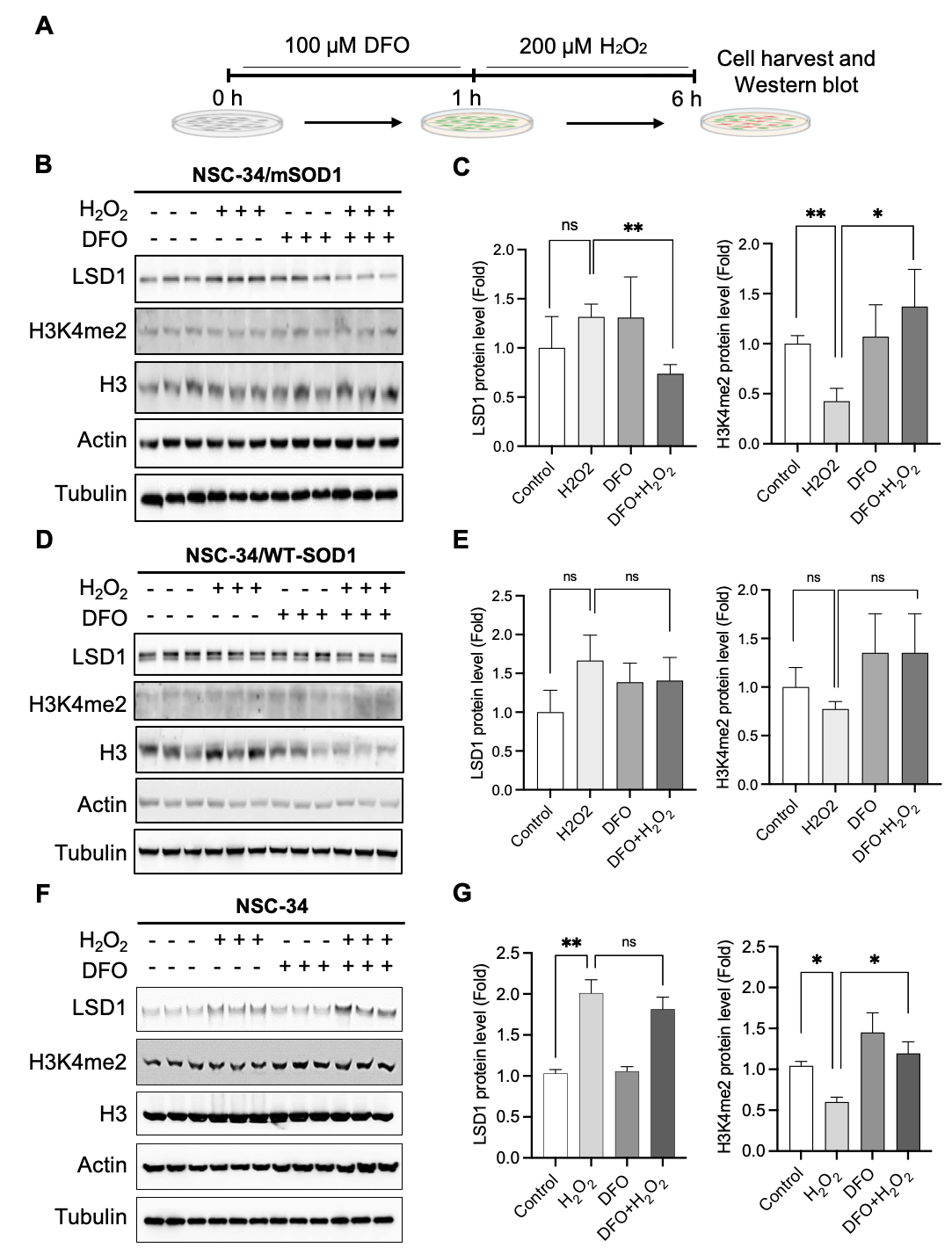


**Figure S2. Antioxidant modulates oxidative stress-induced LSD1-H3K4me2 pathway in a cell line model of ALS. A,** A scheme illustrating procedure of cell experiment. **B,** Western blot analysis showed decrease of LSD1 and elevation of H3K4me2 by antioxidant (deferoxamine: DFO) in hydrogen peroxide (H_2_O_2_)-treated NSC-34/mSDO1 cells. **C,** Densitometry analysis of LSD1 and H3K4m2 levels from Western blot analysis (originated from B). **D,** Western blot analysis showed the LSD1 and H3K4me2 level maintained by DFO in H_2_O_2_-treated NSC-34/WT-SDO1 cells. **E,** Densitometry analysis of LSD1 and H3K4m2 levels from Western blot analysis (originated from D). **F,** Western blot analysis confirmed that DFO elevated the level of H3K4me2 in H_2_O_2_-treated NSC-34 motor neuronal cells. **G,** Densitometry analysis of LSD1 and H3K4m2 levels from Western blot analysis (originated from F). LSD1 and H3K4me2 were normalized to actin and histone H3 (H3), respectively. Significantly different at *p<0.05 and **p<0.01.
